# Evaluation of High-Risk Anatomical Features in Single and Opposite-Sinus Origin Coronary Artery Anomalies Using Coronary Computed Tomography Angiography

**DOI:** 10.3390/diagnostics16152465

**Published:** 2026-08-05

**Authors:** Fatma Durmaz, Ebru Temel Kurt

**Affiliations:** Department of Radiology, School of Medicine, Van Yuzuncu Yıl University, 65400 Van, Türkiye; temelebru469@gmail.com

**Keywords:** coronary artery anomalies, single coronary artery, ACAOS, coronary computed tomography angiography, intramural course, interarterial course

## Abstract

**Objective**: To characterize the anatomical spectrum of a single coronary artery (SCA) and an anomalous coronary artery originating from the opposite sinus of Valsalva (ACAOS) and to assess high-risk features using Coronary computed tomography angiography (CCTA)-based quantitative parameters. **Methods**: A retrospective review of 2513 consecutive CCTA examinations performed between June 2021 and December 2025 was conducted. Patients diagnosed with ACAOS or SCA were included. Coronary origin, course patterns, and morphological characteristics were analyzed. High-risk features—including interarterial course, intramural segment, acute take-off angle, slit-like ostium, intramural length, and interluminal space (ILS)—were quantitatively assessed using multiplanar and curved MPRs. **Results**: Twenty-two patients (0.88%) were identified (ACAOS: 0.64%, *n* = 16; SCA: 0.24%, *n* = 6). All SCA cases showed benign courses. Among ACAOS patients, 62.5% had an interarterial course. In this subgroup, the mean take-off angle was 14.4° ± 4.5°, with universal slit-like ostium and intramural course. Mean intramural length was 9.21 ± 2.00 mm and minimal ILS was 0.87 ± 0.12 mm. **Conclusions**: CCTA enables comprehensive anatomical characterization of morphological features associated with higher risk in patients with ACAOS and SCA. Quantitative assessment of parameters such as the take-off angle, intramural course, slit-like ostium, intramural length, and interluminal space may complement clinical evaluation, although further prospective studies incorporating functional ischemia assessment and clinical outcome data are needed to clarify their clinical significance.

## 1. Introduction

Coronary artery anomalies are a rare but clinically significant subset of congenital cardiovascular conditions, representing the second leading cause of sudden cardiac death (SCD) in young individuals after hypertrophic cardiomyopathy [1,2]. The prevalence of coronary artery anomalies varies significantly by imaging modality; while conventional angiography detects these variations in roughly 1% of cases, coronary computed tomography angiography (CCTA) reveals incidental findings in up to 5.8% of patients [3]. With the increasing use of CCTA, these anomalies are being recognized with increasing frequency.

Anomalous coronary artery originating from the opposite sinus of Valsalva (ACAOS) and single coronary artery (SCA), defined by a single coronary arterial origin from the aorta, constitute rare subtypes within the anomalous aortic origin of a coronary artery (AAOCA) spectrum [4]. Certain anatomical patterns, in conjunction with clinical characteristics, are linked to an elevated risk of myocardial ischemia and SCD in pediatric and physically active young populations, and are consequently regarded as malignant variants [5,6]. Patients with an interarterial course (IAC) are at increased risk of myocardial ischemia and secondary arrhythmias, even in the absence of atherosclerosis, because the anomalous coronary artery may be compressed during physical exertion. The definition of high-risk anatomy extends beyond the classic interarterial variant to include features such as an acute take-off angle, slit-like ostial morphology, and an intramural course—characterized by the proximal vessel sharing the aortic tunica media, which leads to elliptical deformation and luminal narrowing [1,7].

Although invasive coronary angiography (ICA) remains the gold standard for coronary artery assessment, its invasive nature and limited depiction of coronary anomaly anatomy are important limitations. In contrast, CCTA provides comprehensive anatomical evaluation of anomalous coronary arteries in a precise, non-invasive manner [8]. Although quantitative CCTA assessment of individual high-risk anatomical features, including take-off angle, slit-like ostial morphology, intramural course, and interluminal space, has been previously described, studies integrating these established imaging parameters into a comprehensive anatomical characterization of both ACAOS and SCA remain limited. The aim of this study was to characterize the anatomical spectrum of ACAOS and SCA identified at our center by integrating CCTA-derived quantitative assessment of established high-risk anatomical features with anomaly classification and coronary course patterns.

## 2. Materials and Methods

### 2.1. Study Population

This study was initiated following institutional ethics committee approval of the study protocol. Patients who underwent CCTA at our institution between June 2021 and December 2025 were included in the analysis. During this period, a total of 2513 consecutive CCTA examinations were retrospectively reviewed. Patients with imaging findings consistent with ACAOS or SCA were enrolled in the study.

Two patients with congenital heart disease (one with double outlet right ventricle and one with a large ventricular septal defect) were excluded from the analysis. The final study cohort consisted of 22 patients.

We used the STROBE reporting guideline [9] to draft this manuscript, and the STROBE reporting checklist [10].

### 2.2. CCTA Acquisition Protocol

CCTA acquisitions were conducted on a 128-slice multidetector system (Somatom Definition AS+, Siemens Healthineers, Forchheim, Germany). Patients with a resting heart rate greater than 65 beats per minute received oral metoprolol (50–100 mg) at least one hour prior to the examination; additional doses were administered if necessary, up to a maximum total dose of 200 mg. Prior to image acquisition, all patients were instructed in breath-hold techniques under electrocardiographic (ECG) monitoring.

Standard acquisition parameters included a tube voltage of 120 kVp, 0.75 mm slice thickness, and 16 cm detector collimation, extending from the carina to the apex. Tube current was adjusted according to patient body habitus, ranging between 200 and 400 mA. Images were reconstructed using an optimized field of view appropriate for cardiac imaging.

CCTA was performed using a standard ECG-gated, breath-hold protocol. Iodinated contrast material was injected intravenously at a rate of 4–6 mL/s, followed by a saline flush. Scan timing and acquisition parameters were adapted according to heart rate and scanner recommendations to ensure optimal visualization of the coronary arteries.

### 2.3. Image Analysis

All CCTA datasets were transferred to a dedicated workstation (syngo.via VB40 Software, Siemens Healthineers, Forcheim, Germany). All datasets were independently evaluated by two radiologists with 12 and 4 years of radiology experience, respectively, and discrepancies were resolved by consensus.

In all patients, the ostial location and coronary artery course were evaluated using axial CT images and three-dimensional (3D) volume-rendered reconstructions. ACAOS cases were classified based on the sinus of origin of the ectopic ostium [11], including: right coronary artery (RCA) arising from the left coronary sinus; left main coronary artery (LCA) arising from the right coronary sinus; left circumflex (LCx) or left anterior descending (LAD) artery arising from the right coronary sinus; and RCA or LCA (or one of their branches) arising from the non-coronary sinus.

Furthermore, ACAOS cases were categorized into four main course patterns based on the anatomical relationship of the proximal segment of the anomalous artery with the aorta and pulmonary artery [12]. An interarterial course occurs when the coronary artery passes between the aorta and pulmonary artery (malignant course), while a retroaortic course describes an artery running posterior to the aorta, between the aorta and left atrium. In a prepulmonic course, the vessel courses anterior to the pulmonary artery or the right ventricular outflow tract (RVOT), whereas a subpulmonic course is defined as a trajectory beneath the plane of the pulmonary valve annulus.

SCA cases were classified according to the modified Lipton system, taking into account the ostium location (right or left) and the pattern of coronary artery distribution [13]. In this system, the course of the anomalous branch extending into the contralateral perfusion territory is classified into four subtypes according to its relationship with the great vessels: Type A (prepulmonic/anterior), Type B (interarterial/malignant), Type R (retroaortic/posterior), and Type S (septal/subpulmonic).

### 2.4. Morphological and Quantitative Assessment

In all patients, morphological characteristics and quantitative measurements of the proximal segment of the anomalous coronary artery were evaluated using axial source images and multiplanar reconstruction (MPR) techniques.

The take-off angle was measured on curved MPR images as the angle between the tangent to the aortic wall at the coronary ostium and the central axis of the proximal anomalous coronary artery. A take-off angle of <45° was considered acute.

Proximal vessel morphology was analyzed on cross-sectional images oriented orthogonally to the vessel’s long axis. A slit-like ostium was defined when the ratio of the minimal proximal diameter to the distal reference diameter (short-axis/long-axis ratio) demonstrated ≥50% narrowing.

**Figure 1:**
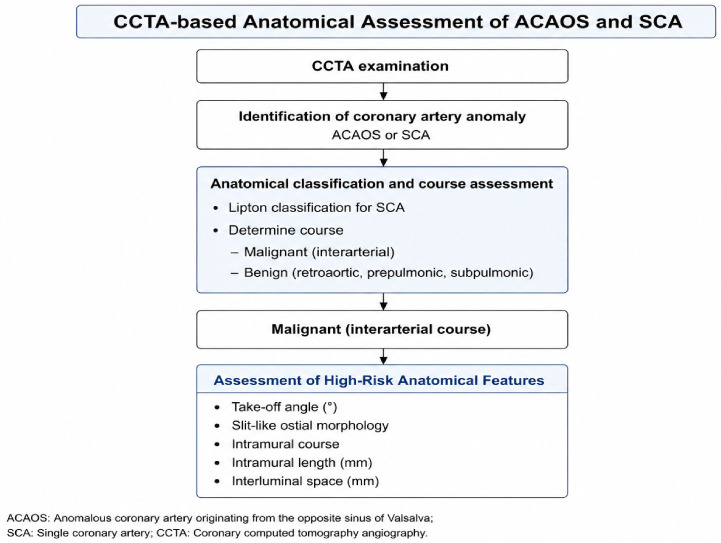
CCTA-based anatomical assessment workflow for ACAOS and SCA.

An intramural course was defined by the absence of protective perivascular fat tissue between the proximal anomalous segment and the aortic lumen, together with a marked reduction or obliteration of the distance between the aortic lumen and coronary lumen (interluminal space, ILS) on serial ostial segment measurements, in accordance with previously published criteria [12,14]. The ILS was measured in millimeters as the shortest distance between the aortic lumen and the coronary lumen along the proximal anomalous segment. Curved MPR images were generated when necessary to optimize measurement. The narrowest ILS value was recorded for analysis. The intramural segment length was measured on curved MPRs from the coronary ostium to the point where the anomalous coronary artery exited the aortic wall, as indicated by the reappearance of perivascular fat. The CCTA-based anatomical assessment workflow is summarized in Figure 1.

### 2.5. Statistical Analysis

Statistical analysis was performed using SPSS version 22.0 (IBM Corp., Armonk, NY, USA). Because of the rarity of these anomalies, all consecutive eligible patients were included in the analysis and no formal sample size calculation was performed. Given the rarity of these anomalies and the limited sample size, data were evaluated using descriptive statistical methods. For the reported prevalence estimates, 95% confidence intervals (CIs) were calculated using the Wilson score method. No inferential statistical comparisons were performed between subgroups. Continuous variables (age, take-off angle, intramural course length, and interluminal distance) were presented as mean ± standard deviation (SD), together with minimum and maximum values (range). Categorical variables (sex, anomaly type, course characteristics, and Lipton classification) were expressed as numbers (*n*) and percentages (%).

## 3. Results

The study comprised 22 patients in whom ACAOS or SCA was detected on CCTA. The mean age of the cohort was 53.7 ± 16.2 years (range: 23–79 years), with 12 females (54.5%) and 10 males (45.5%). Among the 2513 CCTA examinations reviewed during the study period, 22 patients (0.88%) (95% CI: 0.58–1.32%) were diagnosed with ACAOS or SCA. The overall prevalence of ACAOS in the study population was 0.64% (*n* = 16; 95% CI: 0.39–1.03%), whereas the rarer SCA anomaly was identified in 0.24% (*n* = 6; 95% CI: 0.11–0.52%) of cases (Table 1).

The primary clinical indication for CCTA referral was suspected coronary artery disease presenting as chest pain in most patients, whereas dyspnea was the referral indication in a smaller subset. Coronary atherosclerotic plaques within the anomalous vessel were identified in 8 patients, and only one patient (LIIA-SCA) demonstrated hemodynamically significant luminal stenosis requiring surgical revascularization.

**Figure 2:**
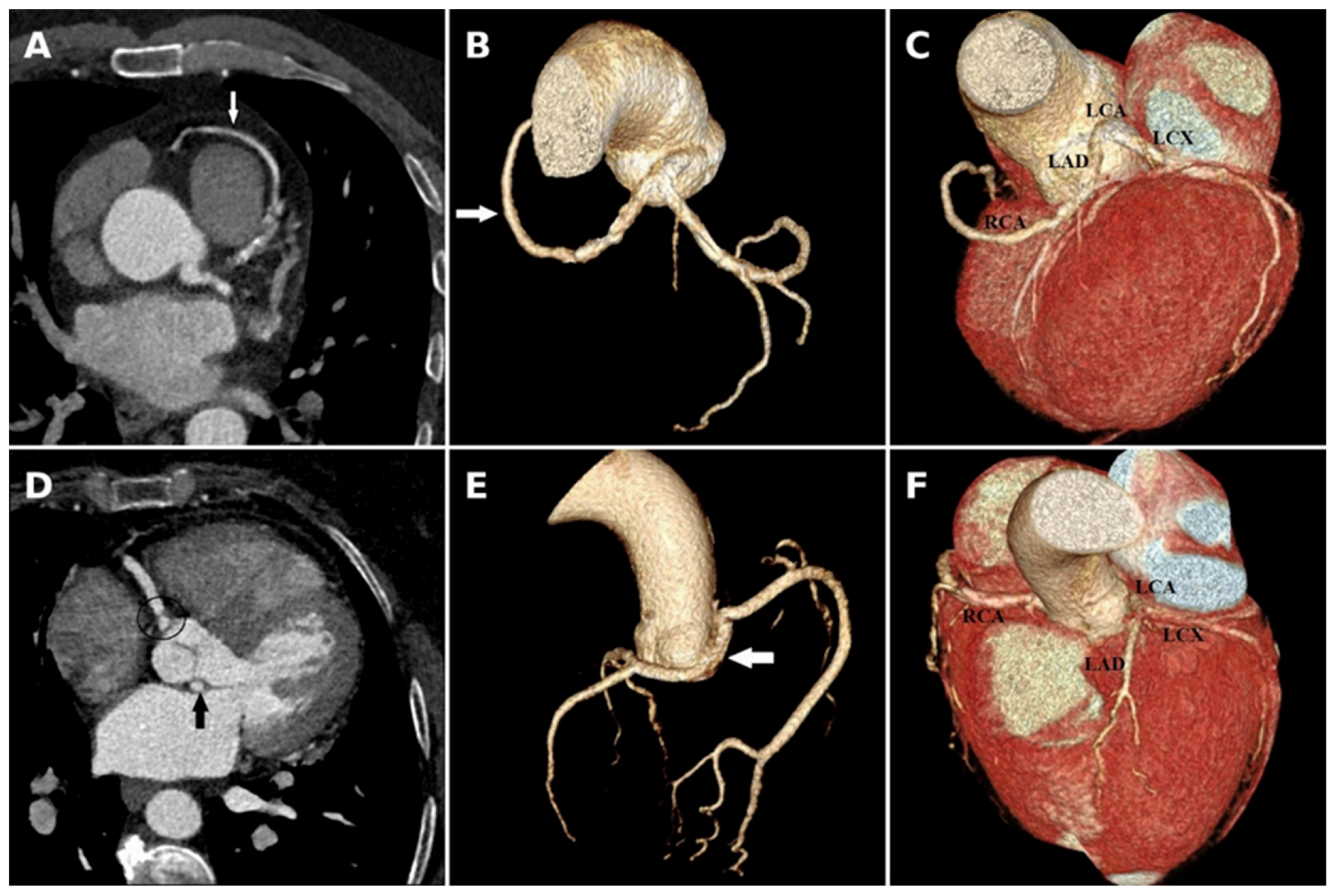
Coronary computed tomography angiography findings in two patients with single coronary artery variants. (**A**–**C**): A 67-year-old man with Lipton type L-II-A single coronary artery. (**A**) Axial CCTA shows the right coronary artery (RCA) originating from the mid left anterior descending artery (LAD) and coursing anterior to the pulmonary artery (arrow), consistent with a prepulmonic course. (**B**) Volume-rendered reconstruction demonstrating the anomalous RCA (arrow). (**C**) Three-dimensional overview with labeled coronary branches. (**D**–**F**): A 71-year-old woman with Lipton type R-II-P single coronary artery. (**D**) Axial CCTA shows the left coronary artery arising from a common trunk with the RCA (circle) and passing posterior to the aorta (arrow), consistent with a retroaortic course. (**E**) Volume-rendered image highlighting the anomalous left coronary origin and course (arrow). (**F**) Three-dimensional reconstruction showing the complete coronary anatomy with labeled vessels.

The six SCA cases were categorized according to the modified Lipton classification system. A retroaortic course was observed in 50% (*n* = 3) of patients, including one case with left sinus origin (L-II P) and two cases with right sinus origin (R-II P). The remaining variants included subpulmonic (R-II S, *n* = 1), prepulmonic (L-II A, *n* = 1), and a variant with normal anatomical distribution (L-I, *n* = 1). All SCA cases demonstrated benign coronary courses (retroaortic, prepulmonic, or subpulmonic). None of the patients exhibited an interarterial course (Lipton type B), nor were compression-related findings associated with acute take-off angles observed (Figure 2).

The 16 ACAOS cases were analyzed according to their anatomical origin and course patterns. The most frequent variant was origin of the RCA from the left sinus of Valsalva (R-ACAOS), observed in 10 patients (62.5%). Other variants included anomalous origin patterns involving the circumflex artery (LCx) (*n* = 4, 25%) and origin of the RCA arising from the non-coronary sinus (*n* = 2, 12.5%) (Table 2).

Risk stratification based on course patterns demonstrated a high-risk interarterial course in 10 ACAOS cases (62.5%). All of these patients were diagnosed with R-ACAOS. The remaining ACAOS cases demonstrated a retroaortic course (*n* = 4, 25%) or a normal/benign course (*n* = 2, 12.5%). No cases of LCA arising from the right sinus of Valsalva (L-ACAOS) were identified.

**Figure 3:**
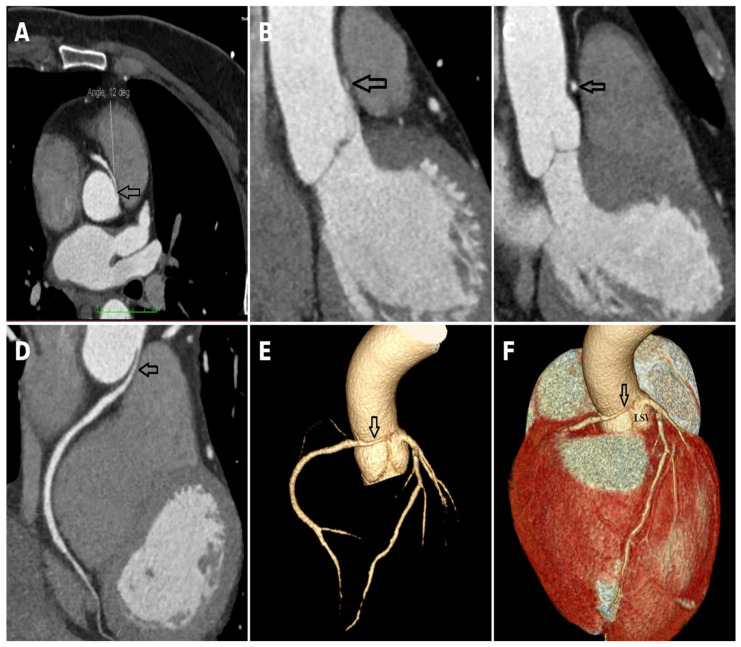
CCTA evaluation of high-risk anatomical features in an anomalous right coronary artery. A 63-year-old man with an anomalous right coronary artery (RCA) originating from the left sinus of Valsalva. (**A**) Axial CCTA demonstrates an acute take-off angle at the RCA origin, measured at 12° (arrow). (**B**) Cross-sectional image of the intramural segment shows an elliptic, laterally compressed lumen between the aorta and pulmonary artery, with 63% luminal narrowing (arrow). (**C**) Cross-sectional view distal to the intramural segment demonstrates restoration of a near-circular luminal configuration of the proximal normal segment (arrow). (**D**) Curved multiplanar reformatted image demonstrates proximal slit-like narrowing of the intramural segment (arrow) and its course between the great vessels. (**E**,**F**) Three-dimensional volume-rendered reconstructions demonstrate the anomalous RCA arising from the left sinus of Valsalva (LSV) with an interarterial/intramural proximal trajectory (arrows).

**Figure 4:**
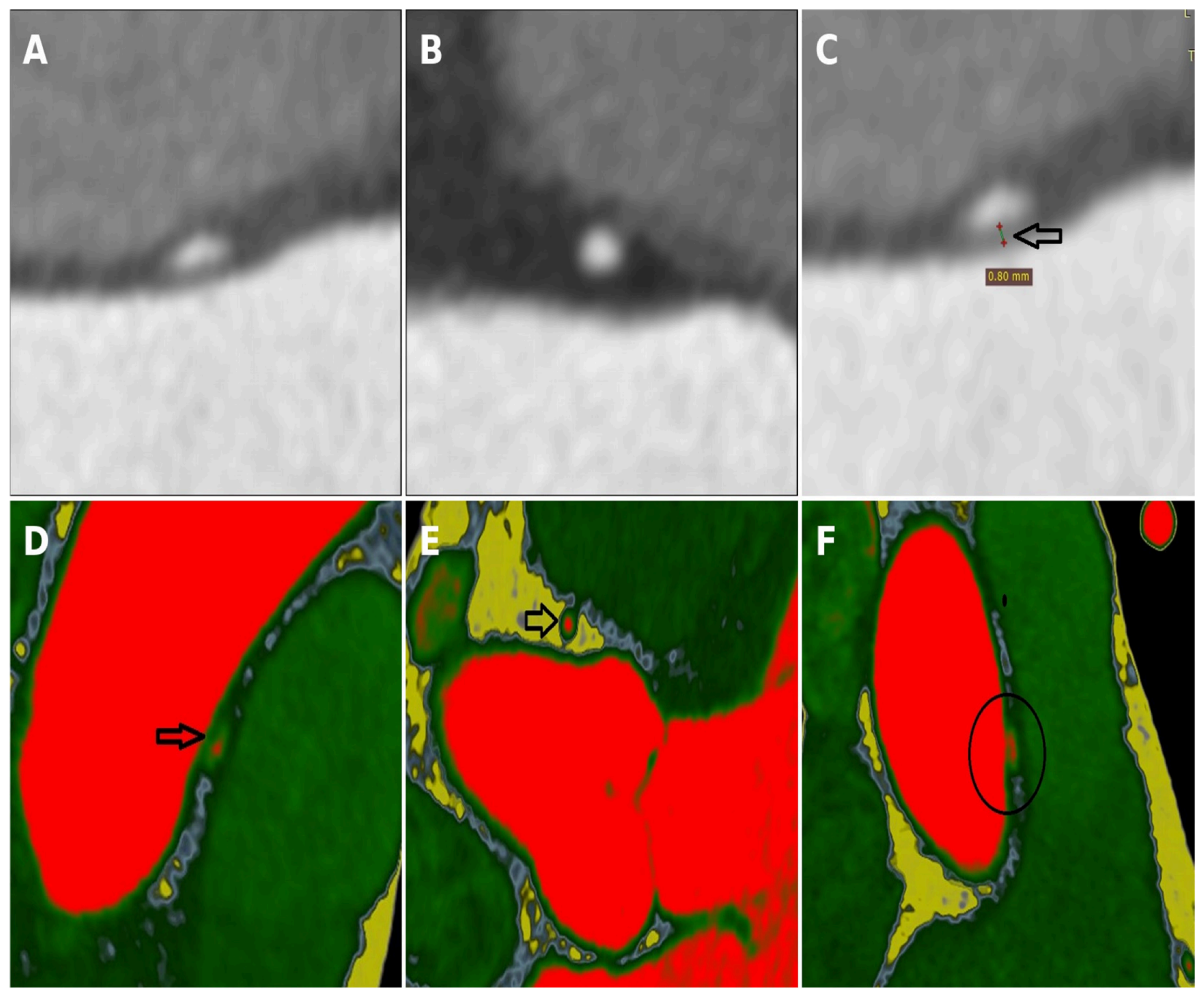
Cross-sectional and color-coded evaluation of the intramural coronary segment on CCTA. (**A**–**C**) High-resolution cross-sectional reformatted images of the proximal intramural segment. (**A**) Elliptic, slit-like lumen configuration consistent with lateral compression within the aortic wall. (**B**) Image obtained immediately distal to the intramural segment demonstrates restoration of a near-circular proximal normal lumen with visible surrounding perivascular fat, indicating exit from the intramural course. (**C**) Direct measurement of the minimal interluminal space (ILS) shows a markedly reduced diameter of 0.80 mm (arrow). (**D**) At the intramural level corresponding to panel A, separation between the coronary and aortic walls cannot be clearly identified (arrow), consistent with an intramural course. (**E**) Distal to the intramural segment, the proximal normal coronary segment (arrow) becomes distinctly separated from the aortic wall, with preserved surrounding tissue planes. (**F**) At the coronary ostium, a slit-like luminal morphology is demonstrated (circle). In the color-coded images (**D**–**F**), contrast-enhanced vascular lumens are displayed in red, adipose tissue in yellow, and other surrounding anatomical structures in green.

Quantitative analysis of high-risk anatomical features was performed in the 10 patients with interarterial course (Figure 3 and Figure 4). The mean take-off angle in this group was 14.4° ± 4.5° (minimum: 10°, maximum: 24°). All interarterial cases demonstrated a slit-like ostial morphology along with an intramural trajectory of the proximal coronary segment. The mean length of the intramural segment was 9.21 ± 2.00 mm (range: 5.6–13.0 mm), and the mean minimal interluminal space (ILS) at the site of maximal compression was 0.87 ± 0.12 mm (range: 0.7–1.1 mm) (Table 3).

## 4. Discussion

This study comprehensively evaluated the anatomical characteristics of SCA and ACAOS cases detected by CCTA at our institution and systematically analyzed the distribution of morphological features considered high risk. The prevalence of ACAOS and SCA was found to be 0.64% and 0.24%, respectively. In all SCA cases, the course of the anomalous vessel was consistent with benign variants, including retroaortic, prepulmonic, and subpulmonic courses, and none of these patients demonstrated high-risk anatomical features. In contrast, a substantial proportion of ACAOS cases (62.5%) exhibited a malignant interarterial course, and all patients in this high-risk group had R-ACAOS. Beyond detecting the presence of the anomaly, CCTA enabled quantitative analysis of critical high-risk parameters—such as take-off angle, intramural segment length, and interluminal distance.

Previous large-scale CCTA studies have shown that the prevalence of SCA and ACAOS ranges from 0.056% to 0.27% [15,16,17] and from 0.78% to 1.7% [12,17,18,19,20], respectively. In ICA-based studies, SCA prevalence ranges from 0.031% to 0.066% [21,22,23], and ACAOS prevalence from 0.26% to 0.58% [23,24]. The prevalences identified in our study (0.24% for SCA and 0.64% for ACAOS) are generally consistent with the published literature and, as expected, are relatively higher than those reported in ICA-based studies, reflecting the superior sensitivity of CCTA for detecting anomalous coronary anatomy.

Consistent with prior reports, the most common ACAOS variant in our cohort was R-ACAOS. All cases with a malignant course belonged to this subgroup. Interarterial coronary anomalies are well known to be associated with SCD, and the presence of an intramural segment has been recognized as the most critical determinant in risk stratification of these anomalies. Miller et al. evaluated the performance of CCTA in patients with surgically confirmed intramural segments and reported a mean take-off angle of 18.3° in patients with intramural course compared with 48.7° in those without. They further demonstrated that slit-like ostium and elliptical vessel morphology showed 100% correlation with surgically proven intramural segments, identifying these features as key discriminators of patients requiring surgical intervention [25]. In our study, the narrow mean take-off angle (14.4° ± 4.5°) observed in all interarterial cases, together with the universal presence of slit-like ostium morphology, closely aligns with these high-risk intramurality criteria. The absence of these features in interarterial cases without intramural segments in Miller’s series demonstrates consistency between our observations and previously reported CCTA findings.

More recently, Koppel et al. introduced a novel quantitative CCTA parameter termed the interluminal space (ILS) for the detection of intramural course in ACAOS [14]. In their analysis of surgically confirmed intramural cases, an aortic-to-coronary luminal distance of less than 0.95 mm identified intramural segments, achieving a sensitivity of 100% and a specificity of 84%. In our study, the mean minimal ILS measured in interarterial cases was 0.87 ± 0.12 mm, consistent with this proposed threshold. Moreover, the combined presence of elliptical vessel morphology along the intramural segment and acute take-off angle reproduces the previously described high-risk anatomical pattern. Although earlier studies by Angelini et al. suggested that the spatial resolution of CT angiography might be insufficient to reliably demonstrate intramurality (tunica media sharing) [26], the millimetric ILS measurements reported by Koppel et al. and observed in our series suggest that contemporary CCTA may facilitate indirect assessment of intramurality.

The clinical relevance of these morphological features is further emphasized by the CCTA-based analysis performed by Marano et al. in an athletic population [27], in which interarterial course, acute take-off angle, slit-like ostium, and intramurality were frequently observed in combination, and this morphological constellation was shown to be decisive in guiding clinical management. Their findings highlight that risk assessment should not be based on isolated anatomical features, but rather on an integrated morphological profile. In our cohort, CCTA demonstrated the coexistence of all high-risk features in interarterial ACAOS cases, allowing detailed anatomical evaluation.

Intramural length is increasingly recognized as an important high-risk anatomical feature in ACAOS and has been incorporated into CCTA-based anatomical assessment in several studies. In pediatric cohorts predominantly comprising R-ACAOS, Kaushal et al. and Balasubramanya et al. measured the intramural segment from the coronary ostium to the point where the artery exited the aortic wall and reported mean intramural lengths of approximately 7.8 mm and 8.7 ± 3.3 mm, respectively [28,29]. Ferraro et al., in a pediatric and young adult cohort, quantified the intramural (oval) segment and reported a mean intramural length of 7.3 ± 3.7 mm overall (7.6 ± 3.2 mm for R-ACAOS and 6.3 ± 5.0 mm for L-ACAOS) [30]. More recently, the adult MuSCAT study, which also consisted predominantly of R-ACAOS patients, measured the intramural segment from the coronary ostium to the point where the ILS reached ≥0.95 mm and reported a median intramural length of 7.3 mm (IQR, 2.7–9.2 mm) [31]. In our cohort, the mean intramural length was 9.21 ± 2.00 mm (range, 5.6–13.0 mm), which is broadly consistent with previously reported CCTA measurements, although direct comparisons should be interpreted in the context of differences in study populations and measurement definitions.

No malignant interarterial course was observed in the SCA group. In one case, an apparent interarterial appearance on axial images was shown on MPR analysis to represent a benign subpulmonic course passing between the RVOT and the aorta. This case illustrates the diagnostic value of CCTA in excluding false-positive interpretations and in differentiating benign variants from malignant entities.

Both the American College of Cardiology/American Heart Association (ACC/AHA) and the European Society of Cardiology (ESC) guidelines emphasize that management of AAOCA should be based not solely on the presence of the anomaly, but on an integrated assessment of symptoms, objective evidence of ischemia, and high-risk anatomical features [4,32]. While the ACC/AHA guidelines prioritize surgical intervention in symptomatic or ischemic patients and recommend a more proactive approach in asymptomatic patients with L-ACAOS, they advocate individualized decision-making in R-ACAOS cases. Similarly, the ESC guidelines underscore the importance of high-risk patterns such as interarterial and intramural course, while noting that anatomical findings alone do not constitute absolute surgical indications in asymptomatic patients, and that management should integrate patient characteristics, functional ischemia assessment, and high-risk anatomical features. Within this framework, the quantitative CCTA parameters evaluated in our study should be interpreted as complementary anatomical findings rather than independent determinants of clinical management. Although the intramural course has been recognized as a key high-risk anatomical feature [14,25], current guidelines do not recommend surgical referral based on any single anatomical parameter or imaging threshold alone [4,32]. The quantitative anatomical findings reported in our study should therefore be interpreted in the context of the limited sample size and require confirmation in larger multicentre cohorts.

Consistent with current guideline recommendations, anatomical assessment should be integrated with functional ischemia evaluation when considering intervention in patients with AAOCA. Stress imaging plays an important role in determining the physiological significance of anatomical abnormalities, while additional functional assessment may be appropriate in selected patients when high-risk anatomy is identified [4,31,32]. Since functional ischemia assessment was not available in our cohort, our findings should be interpreted as anatomical observations rather than indicators of physiological significance.

This study has several limitations. First, this was a retrospective, single-center study with a limited number of cases, reflecting the inherent rarity of ACAOS and SCA, which precluded inferential statistical analyses and the assessment of prognostic associations. In addition, systematic clinical outcomes and long-term follow-up data were not available, preventing correlation between high-risk anatomical features and adverse cardiac events. Another limitation is the absence of functional ischemia assessment, limiting direct evaluation of the physiological significance of the observed anatomical findings. Furthermore, no interobserver or intraobserver reproducibility analysis was performed for the CCTA measurements. Finally, the study population consisted of a selected group of patients undergoing CCTA at a single institution. Consequently, the reported prevalence estimates may not be fully generalizable to other institutions or the general population because of differences in referral patterns and patient selection.

## 5. Conclusions

In conclusion, CCTA enables detailed anatomical characterization of morphological features that have previously been associated with higher risk in patients with ACAOS and SCA. Quantitative evaluation of parameters such as take-off angle, intramural course, slit-like ostium, intramural length, and interluminal space provides a comprehensive anatomical profile that may complement clinical evaluation. Further larger prospective studies integrating functional ischemia assessment and clinical outcome data are needed to better define the clinical significance of these anatomical findings.

## Figures and Tables

**Table 1:** Demographic characteristics and prevalence of ACAOS and SCA.

Variable	Value
Total CCTA examinations	2513
Total ACAOS and SCA cases	22 (0.88%; 95% CI: 0.58–1.32)
Age (years), mean ± SD	53.7 ± 16.2
Female sex, *n* (%)	12 (54.5)
Male sex, *n* (%)	10 (45.5)
ACAOS, *n* (%)	16 (0.64; 95% CI: 0.39–1.03)
SCA, *n* (%)	6 (0.24; 95% CI: 0.11–0.52)

Data are presented as mean ± standard deviation (SD) or number (percentage). CCTA: coronary computed tomography angiography; ACAOS: anomalous coronary artery originating from the opposite sinus of Valsalva; SCA: single coronary artery.

**Table 2:** Anatomical classification and coronary course patterns of ACAOS and SCA.

Anomaly Type	Variable	*N* (%)	Coronary Course	Risk Category
SCA	L-II P/R-II P	3 (50.0)	Retroaortic	Benign
	R-II S	1 (16.7)	Subpulmonic	Benign
	L-II A	1 (16.7)	Prepulmonic	Benign
	L-I	1 (16.7)	Normal course	Benign
ACAOS	R-ACAOS	10 (62.5)	Interarterial	High-risk
	LCx anomalies RCA from NCS	4 (25) 2 (12.5)	Retroaortic Normal course	Benign Benign

Data are presented as number (percentage). ACAOS: anomalous coronary artery originating from the opposite sinus of Valsalva; SCA: single coronary artery; NCS: Non-coronary sinus.

**Table 3:** Quantitative CCTA assessment of high-risk anatomical features in patients with interarterial ACAOS.

Parameter	Value
Acute take-off angle (°), mean ± SD (range)	14.4 ± 4.5 (10–24)
Slit-like ostium, *n* (%)	10 (100)
Intramural course, *n* (%)	10 (100)
Intramural length (mm), mean ± SD (range)	9.21 ± 2.00 (5.6–13.0)
Minimum interluminal space (ILS) (mm), mean ± SD (range)	0.87 ± 0.12 (0.7–1.1)

## Data Availability

The data presented in this study are available on request from the corresponding author due to privacy and ethical restrictions.

## Abbreviations

The following abbreviations are used in this manuscript:

SCA	Single coronary artery
ACAOS	Anomalous coronary artery originating from the opposite sinus
CCTA	Coronary computed tomography angiography
ILS	Interluminal space
AAOCA	Anomalous aortic origin of a coronary artery
SCD	Sudden cardiac death
IAC	Interarterial course
ICA	Invasive coronary angiography
ECG	Electrocardiography
RCA	Right coronary artery
LCA	Left main coronary artery
LCx	Left circumflex artery
LAD	Left anterior descending artery
RVOT	Right ventricular outflow tract
MPR	Multiplanar reconstruction
